# The impact of diabetes on physical and mental health status and patient satisfaction after total hip and knee arthroplasty

**DOI:** 10.1371/journal.pone.0302315

**Published:** 2024-04-24

**Authors:** J. Denise Power, Flaviu Trifoi, Mayilee Canizares, Anthony V. Perruccio, Ajaykumar Shanmugaraj, Rajiv Gandhi, J. Roderick Davey, Khalid Syed, Nizar N. Mahomed, Christian Veillette, Y. Raja Rampersaud

**Affiliations:** 1 Schroeder Arthritis Institute, Krembil Research Institute, University Health Network, Toronto, Ontario, Canada; 2 Arthritis Community Research and Epidemiology Unit (ACREU), Krembil Research Institute, Toronto, Ontario, Canada; 3 Schulich School of Medicine & Dentistry, University of Western Ontario, London, Ontario, Canada; 4 Department of Surgery, Orthopaedics, University of Toronto, Toronto, Ontario, Canada; 5 Institute of Health Policy, Management and Evaluation, Dalla Lana School of Public Health, University of Toronto, Toronto, Ontario, Canada; 6 Faculty of Medicine, University of British Columbia, Vancouver, British Columbia, Canada; Taipei Medical University, TAIWAN

## Abstract

**Objective:**

To assess the impact of diabetes on physical and mental health status, as well as patient satisfaction, one-year following knee and hip total joint arthroplasty (TJA) for osteoarthritis (OA).

**Methods:**

Participants were 626 hip and 754 knee TJA patients. Pre-surgery data were collected on socio-demographics and health status. The 12-item Short Form Health Survey (SF-12) was collected pre- and one year post-surgery, and physical (PCS) and mental component (MCS) summary scores computed. One-year patient satisfaction was also recorded. Four regression models tested the effect of diabetes on: 1) PCS change score; 2) MCS change score; 3) achieving minimal clinically important improvement (MCII) on PCS; and 4) patient satisfaction (‘Somewhat or Very Satisfied’ vs. ‘Somewhat or Very Dissatisfied’). An interaction between surgical joint and diabetes was tested in each model.

**Results:**

Self-reported diabetes prevalence was 13.0% (95% CI: 11.2%-14.7%) and was more common in knee 16.1% (95% CI: 13.4%-18.7%) than hip 9.3% (95% CI: 7.0%-11.5%) patients. In adjusted analyses, change scores were 2.3 units less on the PCS for those with diabetes compared to those without (p = 0.005). Patients with diabetes were about half as likely to achieve MCII as patients without diabetes (p = 0.004). Diabetes was not significantly associated with satisfaction or changes in MCS scores. Diabetes effects did not differ by surgical joint.

**Conclusions:**

Findings support that diabetes has a negative impact on improvements in physical health after TJA. Considering the growing prevalence of OA and diabetes in the population, our findings support the importance of perioperative screening and management of diabetes in patients undergoing TJA.

## Introduction

Total knee (TKA) and hip (THA) arthroplasty are among the most commonly performed elective surgical procedures and are recognized as cost-effective treatments for end-stage osteoarthritis (OA) [1–3]. Representing the largest proportion of direct OA-related costs, demand for these surgeries is large and predicted to greatly increase due, at least in part, to the aging of the population [3–5]. Despite their relatively high effectiveness, TKA and THA are associated with poor outcomes in approximately 20% of patients [6]. Given the large volume of procedures performed, this proportion of poor outcomes is potentially associated with significant societal impacts and costs, in addition to the substantial individual patient impact. To date, considered risk factors for poor total joint arthroplasty (TJA) outcomes have been somewhat limited in scope and findings have varied [7–11].

The global prevalence of diabetes in the general population is estimated to be 9.3% and is projected to continue to increase [12]. In Canada in 2016/2017, 8.8% of Canadians were reported to have diabetes [13]. However, diabetes and OA share risk factors such as older age and obesity, and diabetes is more common in OA patients than in the general population [14]. A large 2015 meta-analysis estimated a diabetes prevalence of 14.4% among OA patients and determined that the risk of prevalent diabetes was 1.4 times greater in the OA than non-OA population [15].

Diabetes has been associated with higher rates of post-TJA complications, such as infection and aseptic loosening [16,17]. However, relatively few studies have explicitly considered the impact of diabetes on TJA patient-reported outcomes (PROMs), with conflicting findings [18–23]. Studies have generally been limited to TKA patients and have varied in terms of specific outcomes considered and how these outcomes are assessed. Most have examined the impact of diabetes on status scores at various post-surgical time points, rather than relative measures such as change scores or achieving a minimally clinically important improvement (MCII) in score, which take into account a patient’s pre-surgical status. Additionally, some studies have omitted or only controlled for a limited number of potentially important patient factors in analyses. These methodological limitations make interpreting and comparing available findings with respect to diabetes impact challenging.

Understanding the impacts of diabetes on TJA patient-reported outcomes may have implications for pre- or peri-operative patient management. This is particularly salient at this time given the large and growing number of individuals with OA and diabetes. The purpose of this study was to assess the impact of self-reported comorbid diabetes on patient-reported physical and mental health summary outcomes one year following TKA and THA surgery for OA. Absolute change scores and achieving a MCII were included as study outcomes, and a range of patient factors were considered. The impact of diabetes on patient satisfaction with surgery was also examined.

## Methods

This is a retrospective analysis of data from a prospective cohort study (Longitudinal Evaluation in the Arthritis Program–LEAP-OA) conducted at one of Canada’s largest academic health centres by TJA volume, Toronto Western Hospital in Toronto, Canada. Patients with end-stage hip or knee OA scheduled for TJA were consecutively recruited into the cohort from November 18 2013 to March 12 2018. Eligibility criteria included ≥35 years of age and the ability to read and comprehend English. Individuals undergoing revision procedures and those with post-traumatic or inflammatory types of arthritis were excluded. All cohort participants were included in the current analysis, totaling 626 hip and 754 knee OA patients scheduled for unilateral TJA. Data were accessed for the presented analyses on Nov 3 2022. The study was approved by the University Health Network Research Ethics Board (16–5759). Written informed consent was obtained from all patients.

### Data collection

Patients completed a pre-surgery questionnaire within the 3 weeks prior to surgery and follow-up questionnaire at their 12-month post-surgical clinical visit.

#### Socio-demographic variables

Data were collected on socio-demographic characteristics in the pre-surgery questionnaire including sex, age and highest level of education (categorized as post-secondary vs. high school or less).

#### Pre-surgery health characteristics variables

A comorbidity-related count variable was derived from yes/no responses to an extended list of 19 conditions, excluding diabetes, based on the American Academy of Orthopedic Surgeon’s Comorbidity scale [24], and categorized as 0–1, 2–3 and 4+ conditions. A separate variable for diabetes status (present vs. absent) was also created.

Data on measured height and weight were used to compute body mass index (BMI), categorized as normal (18.5–24.9 kg/m2), overweight (25–29.9kg/m2), and obese (30+kg/m2) [25]. Participants indicated on a homunculus diagram any joints/sites that were affected by arthritis and were painful on most days for at least a month. In addition to a variable representing the surgical joint (hip vs. knee), a summed count score of left and right affected joints was derived, excluding the surgical joint.

Neuropathic pain symptoms were assessed using the painDETECT questionnaire (PD-Q) [26], which consists of 9 items that evaluate pain quality, pattern and radiation. Possible scores range from -1 to 38, with higher scores indicating more neuropathic-like symptoms. The PD-Q has been used in a number of clinical populations, including knee OA and other musculoskeletal conditions, with favourable reliability and validity [26]. Sensitivity, specificity and predictive accuracy of 80–84% were determined in a heterogeneous group of pain patients relative to pain physicians’ clinical assessments [27]. For the current study, patients were prompted to consider their hip or knee pain, as appropriate. Scores were dichotomized as unlikely or possibly versus likely neuropathic pain (scores: ≤18 vs. ≥19) [27].

#### Pre- and post-surgery physical and mental health

The physical (PCS) and mental component (MCS) summary scores derived from the 12-item Short Form Health Survey (SF-12) [28] were computed pre- and 12 months post-surgery. The PCS and MCS are generic measures of overall physical and mental health, with possible scores on each ranging from 0–100; lower scores indicate poorer health status. Changes in scores from pre-surgery to 12-months post-surgery were computed. For regression analyses, a variable reflecting quartiles of the pre-surgical PCS and MCS scores was included in the models for PCS and MCS scores, respectively [29].

As TJA is intended to target physical health components, the percentage of patients achieving a minimally clinically important improvement (MCII) in PCS scores was also determined. MCII estimates reported for the PCS for hip and knee TJA patients are 4.6 [30] and 4.3 [31], respectively.

#### Surgical satisfaction

Satisfaction was determined by patient response to the following question on 12-month post-surgical questionnaire: “How satisfied are you with the results of your surgery?” Responses were dichotomized as ‘Somewhat or Very Satisfied’ vs. ‘Somewhat or Very Dissatisfied’

### Analyses

All analyses were conducted using SAS version 9.4. Diabetes prevalence estimates were calculated for the sample overall and by surgical joint. Descriptive statistics were generated for all pre-surgery socio-demographic and health variables; means and standard deviations for continuous variables, frequencies and percentages for categorical variables. These were generated overall and separately for patients with and without diabetes. Differences between these latter groups were assessed using t-tests and chi-square tests, as appropriate. Mean pre- and post-surgical PCS and MCS scores were similarly computed and compared, including change scores and the percentage of patients achieving surgical response defined as achieving a MCII on the PCS. The percentage of patients reporting they were ‘Somewhat or Very Satisfied’ was also computed for the patient sample overall and by diabetes status.

To examine the association between diabetes status (independent variable of interest) and the patient-reported outcomes at 12 months following surgery, four multivariable regression models were estimated controlling for the noted pre-surgery variables (surgical joint, age, sex, education, BMI, comorbidity, neuropathic pain score, pre-surgical PCS or MCS score). The models were:

1. a linear regression model with PCS change score as the dependent variable;
2. a linear regression model with MCS change score as the dependent variable;
3. a logistic regression model with PCS MCII surgical response status (yes vs. no) as the dependent variable;
4. a logistic regression model with surgical satisfaction as the dependent variable.

To test whether the effect of diabetes status on a given response outcome varied for hip and knee TJA patients, an interaction between surgical joint and diabetes status was tested in each of the four models.

## Results

Of the 1380 patients included in the study, 179 reported diabetes for an overall prevalence of 13.0% (95% CI: 11.2%-14.7%). Diabetes was more common among knee than hip patients. Prevalence estimates in these groups were 16.1% (95% CI: 13.4%-18.7%) and 9.3% (95% CI: 7.0%-11.5%), respectively.

Characteristics of the patient sample prior to surgery are presented in Table 1, overall and by diabetes status. Mean patient age was 65.5 years with patients with diabetes being slightly older on average (66.7 years vs. 65.4 years; p = 0.038). Both groups were approximately 55% female. There was a significant difference in BMI, with 60.9% of patients with diabetes being obese compared to 42.5% of patients without diabetes. Patients with diabetes also had a greater number of comorbid conditions, with almost 25% of these patients reporting 4 or more comorbid conditions, compared to only 12% of patients without diabetes. Neuropathic pain-like symptoms (painDETECT scores) and symptomatic joint counts were similar for both groups.

**Table 1:** Pre-surgery sample characteristics, overall and by diabetes status.

Variable	Mean (SD) or Frequency (%)			p-value*
	Overall Sample (N = 1380)	With Diabetes (N = 179)	Without Diabetes (N = 1201)	
**Surgical joint** Knee Hip	754 (54.6%) 626 (45.4%)	121 (67.6%) 58 (32.4%)	633 (52.7%) 568 (47.3%)	**<0.001**
**Sex** Female	758 (54.9%)	97 (54.2%)	661 (55.0%)	0.832
**Age**	65.5 (9.3)	66.7 (7.6)	65.4 (9.5)	**0.038**
**Education**				**0.021**
Post-secondary	952 (70.7%)	108 (63.2%)	844 (71.8%)	
**BMI** Normal	272 (19.7%)	21 (11.7%)	251 (20.9%)	
Overweight	489 (35.4%)	49 (27.4%)	440 (36.6%)	**<0.001**
Obese	619 (44.9%)	109 (60.9%)	510 (42.5%)	
**Comorbidity Count** 0–1 2–3 4+	697 (50.7%) 490 (35.7%) 187 (13.6%)	50 (28.3%) 86 (48.6%) 41 (23.2%)	647 (54.1%) 404 (33.8%) 146 (12.2%)	**<0.001**
**Neuropathic pain score (-1 to 38)** Likely	164 (12.7%)	27 (16.4%)	137 (12.2%)	0.130
**Symptomatic joint count**	2.8 (3.1)	2.7 (3.4)	2.8 (3.1)	0.787

Short Form Physical and Mental Component Scores (SF PCS and MCS, respectively) pre- and post-surgery are presented for patients with and without diabetes in Table 2, with lower scores indicating poorer health status. Overall mean SF PCS and MCS scores prior to surgery were 32.3 (95% CI: 31.8–32.7) and 51.7 (95% CI: 51.1–52.2), respectively, with no significant differences between patients with and without diabetes. At 12 months post-surgery, patients with diabetes had experienced significantly less improvement in their PCS scores and had significantly worse SF PCS scores than patients without diabetes at this time point (12 month PCS scores: 40.0 vs. 43.9; p<0.001). This translated to a lower percentage of patients with diabetes achieving a surgical response based on joint-specific MCII criteria for the PCS (60.7% vs. 74.8%; p**<**0.001). In contrast to these PCS score differences after surgery, there were no significant differences in changes in MCS scores or mean MCS scores at 12 months between patients with and without diabetes. Both groups experienced a 1.2 unit improvement in MCS scores on average (Table 2) and additionally reported similarly high rates of surgical satisfaction, with approximately 93% of patients with and without diabetes reporting they were somewhat or very satisfied with the results of their surgery.

**Table 2:** Pre- and post-surgery PCS and MCS scores and surgical satisfaction, by diabetes status.

	Mean or Percent (95% Confidence Interval)			p- value**
	Overall Sample	With Diabetes	Without Diabetes	
**Pre-surgery**				
PCS Mean score	32.3 (31.8, 32.7)	31.5 (30.2, 32.9)	32.4 (31.8, 32.9)	0.280
MCS Mean score	51.7 (51.1, 52.2)	51.2 (49.5, 52.9)	51.7 (51.1, 52.3)	0.537
**12 months post-surgery: PCS**				
Mean score	43.4 (42.8, 44.0)	40.0 (38.2, 41.7)	43.9 (43.3, 44.5)	**<0.001**
Mean change score	11.1 (10.5, 11.7)	8.3 (6.7, 10.0)	11.5 (10.8, 12.1)	**0.001**
Percent MCII responders	73.1% (70.5%, 75.6%)	60.7% (52.7%, 68.7%)	74.8% (72.2%, 77.5%)	**<0.001**
**12 months post-surgery: MCS** Mean score Mean change score	53.0 (52.4, 53.5) 1.2 (0.6, 1.8)	52.0 (49.5, 52.9) 1.2 (-0.4, 2.8)	53.1 (52.5, 53.6) 1.2 (0.6, 1.8)	0.237 0.973
**12 months post-surgery: Surgical satisfaction** Percent ‘Somewhat or Very Satisfied’	92.7% (91.3%, 94.2%)	92.9% (88.8%, 96.9%)	92.7% (91.1%, 94.3%)	0.946

*Scores on the SF PCS and MCS range from 0 to 100, with lower scores indicating greater severity.

**Comparing those with vs. without diabetes.

Results from the linear regression analyses with PCS and MCS change scores as dependent variables are presented in Tables 3 and 4, respectively. On average at 12 months post-surgery, patients with diabetes improved by 2.3 units less on the PCS than those without diabetes (p = 0.005). Hip patients had a 3.2 unit greater improvement in PCS scores than knee patients (p<0.001). Increasing age was significantly associated with less improvement in PCS scores. Being obese vs. normal weight, and having 4+ vs. 0–1 comorbid conditions, were each associated with an approximately 2 units less improvement in PCS scores. Sex and neuropathic pain scores were not associated with changes in PCS scores. A greater number of symptomatic joints was associated with less improvement in PCS scores, while a higher (better) pre-surgical MCS score was significantly associated with greater improvement in PCS scores after surgery. Patients who had the worst PCS scores pre-surgery (i.e. quartile 1) experienced the greatest amount of improvement after surgery; average improvement of 12.7 units more than those in the top (best) pre-surgery quartile.

**Table 3:** Associations with 12-month SF PCS change scores*.

Variable	Outcome: PCS change	
	Estimate (95% CI)	p-value
**Diabetes** (yes vs. no)	**-2.29 (-3.87, -0.70)**	**0.005**
**Surgical Joint** (Hip vs. Knee)	**3.16 (2.1, 4.2)**	**< .001**
**Age**	**-0.13 (-0.19, -0.07)**	**< .001**
**Sex** (Female vs. Male)	-0.009 (-1.08, 1.06)	0.987
**Education** Post-secondary vs. ≤highschool	0.79 (-0.38, 1.96)	0.187
**Body mass index** (vs. normal)		
Overweight	-0.70 (-2.16, 0.76)	0.349
Obese	**-2.05 (-3.54, -0.56)**	**0.007**
**Comorbidity count** (vs. 0–1)		
2–3	-0.78 (-1.92, 0.36)	0.182
4+	**-1.98 (-3.70, -0.27)**	**0.023**
**Neuropathic pain score** (painDetect) Likely vs. unlikely or possibly	-1.53 (-3.19, -0.14)	0.072
**Symptomatic joint count**	**-0.40 (-0.58, -0.22)**	**< .001**
**Pre-surgical SF PCS quartile** (vs. quartile 4 (best))		
Quartile 1 (worst)	**12.66 (11.14, 14.17)**	**< .001**
Quartile 2	**9.97 (8.50,11.45)**	**< .001**
Quartile 3	**5.19 (3.76, 6.62)**	**< .001**
**Pre-surgical MCS score**	**0.16 (0.11, 0.21)**	**< .001**

*An additional model was estimated with an interaction between surgical joint and diabetes status: PCS change–interaction p = 0.920. Data in the table above are from the model that omits the insignificant interaction.

**Table 4:** Associations with 12-month SF MCS change scores*.

Variable	Outcome: MCS change	
	Estimate (95% CI)	p-value
**Diabetes** (yes vs. no)	-0.07 (-1.54, 1.41)	0.926
**Surgical Joint** (Hip vs. Knee)	0.82 (-0.17, 1.80)	0.105
**Age**	0.02 (-0.03, 0.08)	0.405
**Sex** (Female vs. Male)	0.52 (-0.48, 1.52)	0.309
**Education** Post-secondary vs. ≤highschool	0.86 (-0.23, 1.94)	0.122
**Body mass index** (vs. normal)		
Overweight	1.03 (-0.34, 2.39)	0.140
Obese	**1.42 (0.03, 2.80)**	**0.046**
**Comorbidity count** (vs. 0–1)		
2–3	-0.23 (-1.29, 0.84)	0.675
4+	**-1.88 (-3.47, -0.29)**	**0.021**
**Neuropathic pain score** (painDetect) Likely vs. unlikely or possibly	-1.32 (-2.86, 0.23)	0.094
**Symptomatic joint count**	-0.08 (-0.24, 0.09)	0.367
**Pre-surgical SF MCS quartile** (vs. quartile 4 (best))		
Quartile 1 (worst)	**16.08 (14.68, 17.48)**	**< .001**
Quartile 2	**9.96 (8.60, 11.31)**	**< .001**
Quartile 3	**4.59 (3.26, 5.92)**	**< .001**
**Pre-surgical PCS score**	**0.09 (0.04, 0.15)**	**0.001**

*An additional model outcome was estimated with an interaction between surgical joint and diabetes status: MCS change interaction p = 0.740. Data in the table above are from the model that omits the insignificant interaction.

Having diabetes was not significantly associated with change in MCS scores (Table 4). However, analogous to the finding for PCS change scores, patients with the worst pre-surgical MCS scores experienced the greatest improvements in MCS scores after surgery. Having a better pre-surgical physical health score was associated with significantly greater improvement in mental health status. Patients with more comorbid conditions experienced significantly less improvement in MCS scores than those with ≤1 comorbid condition. In contrast to findings relating to PCS change score, patients who were obese experienced significantly greater improvements in MCS scores after surgery than those who were of normal BMI (p = 0.046).

Analyses did not support that the effects of diabetes status on either PCS or MCS change scores varied for THA and TKA patients. Interaction terms for surgical joint and diabetes status were insignificant when added to the PCS (p = 0.920) and MCS (p = 0.740) change regression models.

The results of logistic regression analyses for predicting surgical response based on achieving joint-specific MCIIs on the PCS are presented in Table 5. Findings are similar as those for PCS change scores. Patients with diabetes were half as likely to achieve MCII as patients without diabetes and this effect did not vary between hip and knee patients (interaction term p = 0.603).Hip patients were 1.6 times more likely to achieve an MCII than knee patients. Increasing age, joint count and being obese vs. normal weight were each associated with reduced odds of surgical response, while better pre-surgical mental health status was significantly associated with increased odds of response. Patients who had pre-surgical PCS scores in the lowest (worst) quartile were 6.3 times more likely to achieve MCII than those with pre-surgical scores in the highest (best) quartile.

**Table 5:** Associations with 12-month response on the SF PCS based on MCII of 4.6 for hip TJA patients and 4.3 for knee TJA patients*.

Variable	Odds Ratio (95% CI)	p-value
**Diabetes** (yes vs. no)	**0.54 (0.36, 0.82)**	**0.004**
**Surgical Joint** (Hip vs. Knee)	**1.61 (1.19, 2.19)**	**0.002**
**Age**	**0.98 (0.96, 0.99)**	**0.008**
**Sex** (Female vs. Male)	1.30 (0.96, 1.77)	0.095
**Education** Post-secondary vs. ≤highschool	1.02 (0.74, 1.42)	0.888
**Body mass index** (vs. normal)		
Overweight	0.92 (0.60, 1.42)	0.716
Obese	**0.59 (0.38, 0.91)**	**0.017**
**Comorbidity count** (vs. 0–1)		
2–3	0.97 (0.70, 1.35)	0.861
4+	0.71 (0.44, 1.13)	0.144
**Neuropathic pain score** (painDetect) Likely vs. unlikely or possibly	0.77 (0.48,1.21)	0.252
**Joint count**	**0.92 (0.88, 0.97)**	**0.001**
**Baseline SF PCS quartile** (vs. quartile 4 (best))		
Quartile 1 (worst)	**6.28 (3.95, 9.97)**	**< .001**
Quartile 2	**5.13 (3.32, 7.93)**	**< .001**
Quartile 3	**2.29 (1.57, 3.33)**	**< .001**
**Pre-surgical MCS score**	**1.02 (1.01, 1.04)**	**0.006**

*An additional model was estimated with an interaction between surgical joint and diabetes status: For the interaction, p = 0.603. Data in the table above are from the model that omits this insignificant interaction.

Based on results from the multivariable logistic regression model examining satisfaction (Table 6), hip patients were 2.2 times more likely than knee patients to report they were satisfied with the results of their surgery. All other covariates in this model, including diabetes status (p = 0.777), were not significantly associated with satisfaction, and findings for diabetes did not vary between hip and knee patients (interaction term p = 0.608).

**Table 6:** Associations with surgical satisfaction 12-months after hip and knee TJA.

Variable	Odds Ratio (95% CI)	p-value
**Diabetes** (yes vs. no)	1.11 (0.54, 2.31)	0.777
**Surgical Joint** (Hip vs. Knee)	**2.16 (1.28, 3.65)**	**0.004**
**Age**	0.99 (0.96, 1.01)	0.280
**Sex** (Female vs. Male)	1.29 (0.79, 2.13)	0.312
**Education** Post-secondary vs. ≤highschool	1.18 (0.71, 1.98)	0.523
**Body mass index** (vs. normal)		
Overweight	0.74 (0.37, 1.49)	0.399
Obese	1.50 (0.70, 3.20)	0.296
**Comorbidity count** (vs. 0–1)		
2–3	0.97 (0.56, 1.70)	0.927
4+	0.69 (0.34, 1.40)	0.308
**Neuropathic pain score** (painDetect) Likely vs. unlikely or possibly	0.56 (0.30, 1.06)	0.073
**Joint count**	1.04 (0.96. 1.13)	0.295
**Pre-surgical PCS score**	**1.05 (1.02, 1.08)**	**0.004**
**Pre-surgical MCS score**	**1.04 (1.02, 1.06)**	**0.001**

*An additional model was estimated with an interaction between surgical joint and diabetes status: For the interaction, p = 0.608. Data in the table above are from the model that omits this insignificant.

## Discussion

Our study of a cohort of TJA patients found that diabetes was associated with less improvement in physical health status one year after hip or knee TJA, and a reduced likelihood of meeting MCII criteria for surgical response based on the SF-12 PCS. These findings persisted even after controlling for a range of factors, including pre-surgical physical and mental health status, neuropathic-like pain symptoms, other comorbidities and obesity. Findings are particularly important given the aging of the population and the growing number of individuals with OA and diabetes.

Our prevalence estimates for diabetes among knee and hip TJA patients of 16.1% (95% CI: 13.4%-18.7%) and 9.3% (95% CI: 7.0%-11.5%) respectively, are in line with the joint-specific estimates reported in Louati et al’s [15] meta-analysis of 17.2±2.0% and 12.3±1.3%. Our inclusion of hip patients is important, as there has been relatively little study of the impact of diabetes on hip TJA outcomes. Our findings were consistent for hip and knee patients across the considered outcomes. Lenguerrad et al [32] also included hip and knee TJA patients in their study examining the impact of self-reported diabetes on WOMAC pain and function scores following surgery. However, they were unable to assess whether their findings differed by surgical joint group. They did find that TJA patients with diabetes had scores reflecting poorer outcomes one year post-surgery, but unlike in the present study, these differences were not evident after controlling for BMI and number of comorbidities. It is unclear to what extent our disparate findings are influenced by the use of differing outcome measures and the range of considered covariates. Further, sample size in the Lenguerrad et al [32] study was less than half the size of our sample and this may also be a contributing factor to the differential findings.

Teo [19] and Clement [18] examined outcomes more similar to those in our study, although only for TKA patients. These studies agreed with ours in that they found no impact of diabetes on surgical satisfaction. Rates of surgical satisfaction after TJA are generally reported to be high [9], despite the estimated 20% or more of patients that experience poor pain and function outcomes [6]. We found that 93% of patients, irrespective of diabetes status, were very or somewhat satisfied with the results of their surgery. Interestingly, this was the case despite the fact that 40% of patients with diabetes and 25% of those without did not meet MCII criteria on the PCS.

Diabetes has a number of adverse effects on the musculoskeletal system that could be relevant for TJA physical outcomes, including poorer wound healing [33] and muscle strength [34], reduced joint mobility [35], and impaired bone quality [36]. However, available research on the impact of diabetes on physical health outcomes after TKA has varied. Teo et al [19] found no difference in mean PCS scores post-surgery, but did not perform any statistical adjustment for factors such as BMI or comorbidity. Similar to our findings, Brock et al [37] determined that diabetes was associated with poorer improvements in PCS after TKA even after controlling for pre-surgery factors, while Zhang [21] and Clement’s [18] findings differed. Other studies [20–23] have also reported conflicting findings, using a variety of methodologies and physical outcome measures. There is clearly a need for additional work on the impact of diabetes on TJA outcomes, incorporating a range of PROMs and consistently including multivariable adjustment to better facilitate study comparison. A range of potentially important factors, such as the presence of neuropathic-like pain symptoms or other symptomatic joints, which may impact joint-specific and generic outcomes following surgery, have not previously been included [38–40]. Additionally, it is important to consider surgical success criteria, such as an MCII, in order to understand if any identified statistical differences represent clinically meaningful differences. We did not identify any other studies that modelled this outcome. It is interesting to note from our results that pre-surgical scores on a given outcome measure were strongly predictive, such that patients with the worst scores before surgery were the most likely to experience the greatest improvements and to achieve MCII. The importance of accounting for pre-surgical symptom levels is increasingly being recognized in the literature [29] and has led to the development of surgical success criteria, for example for WOMAC scores, that vary by pre-surgical symptoms scores [41–43]. We are unaware of any such criteria for the SF PCS.

We found that MCS scores pre- and post-surgery were the same for patients with and without diabetes, with scores improving after surgery only minimally (1.2 units), and the presence of diabetes was not related to changes in MCS scores in the adjusted regression model. This finding is consistent with two similar TKA studies that also utilized multivariable analytical techniques [21,37]. In contrast, Teo [19] and Clement [18] found that although patients with diabetes had poorer mental health scores based on the MCS pre-surgery, they did not after surgery, indicating they experienced greater improvements in mental health. This was true even after multivariable adjustment in Clement’s [18] study. Diabetes has been reported to be associated with poorer mental health. The prevalence of depression is estimated to be two to three times higher in people with diabetes than in those without diabetes [44]. This difference in mental health status by diabetes status was not evident in our sample, at least not as captured by the SF MCS, and may have influenced our findings. Additional research considering the impact of diabetes on more specific measures of mental health after TJA such as depression and anxiety may be helpful in further elucidating these relationships. It may also be that differences in patient populations between studies, or in diabetes or mental health care available or provided, may have influenced study results.

In addition to our consideration of MCII and our adjustment for pre-surgical scores in multivariable analyses that included a range of variables, additional strengths of our study include its relatively large sample size and explicit testing of whether diabetes effects varied between hip and knee patients. Our findings are generalizable to patients who could read and comprehend English. Approximately 10% of patients approached to be included in our study were excluded due to a language barrier. Although the prevalence of diabetes in these patients may differ from those included in our study, it is unlikely that the impact of diabetes on TJA outcome would differ among these patients. However, additional work is needed to confirm this hypothesis. Although the accuracy of self-reported diabetes has generally been found to be high [45,46], some patients may have been misclassified. We were also unable to distinguish between type 1 and type 2 diabetes. Given the relative prevalence of these conditions, it is likely that our results are most generalizable to patients with type 2 diabetes. Patients with undiagnosed diabetes may have been included in our non-diabetes group, with the potential effect of biasing estimates towards the null (i.e. underestimating the effect of diabetes on outcome). We also did not have access to data on specific measures of diabetes control such as blood glucose or HbA1c levels pre- or post-surgery. It may be that there is a dose-response effect between these measures and surgical outcomes [37]. Finally, duration of diabetes, as well as longer-term and time-varying measures of glycemic control, may also be relevant to consider in future studies.

While patients with diabetes in our study reported similarly high rates of satisfaction with TJA than patients without diabetes, we found that the presence of diabetes had a negative impact on improvements in physical health after surgery, such that patients with diabetes were less likely to meet clinically important improvement criteria. This finding was evident even after controlling for comorbidity, obesity and other factors, and did not vary by surgical joint. Given the rising volume of TJAs for OA and the high prevalence of diabetes, the variation in literature findings with respect to diabetes impact warrants additional research to confirm and further elucidate findings. Considering the growing prevalence of OA and diabetes in the population, our findings may have implications for peri-operative patient management aimed at mitigating modifiable risk factors. In turn, this may have the potential to improve overall TJA surgical success rates and reduce costs associated with the growing numbers of these procedures, particularly for those with comorbid diabetes.
